# Identification of common and distinct origins of human serum and breastmilk IgA1 by mass spectrometry-based clonal profiling

**DOI:** 10.1038/s41423-022-00954-2

**Published:** 2022-11-29

**Authors:** Kelly A. Dingess, Max Hoek, Danique M. H. van Rijswijk, Sem Tamara, Maurits A. den Boer, Tim Veth, Mirjam J. A. Damen, Arjan Barendregt, Michelle Romijn, Hannah G. Juncker, Britt J. van Keulen, Gestur Vidarsson, Johannes B. van Goudoever, Albert Bondt, Albert J. R. Heck

**Affiliations:** 1 Biomolecular Mass Spectrometry and Proteomics, Bijvoet Center for Biomolecular Research and Utrecht Institute for Pharmaceutical Sciences, University of Utrecht, Padualaan 8, Utrecht, 3584 CH The Netherlands; 2 Netherlands Proteomics Center, Padualaan 8, Utrecht, 3584 CH The Netherlands; 3 Amsterdam UMC, Vrije Universiteit, University of Amsterdam, Emma Children’s Hospital, Amsterdam Reproduction & Development Research Institute, Department of Pediatrics, Amsterdam, the Netherlands; 4 Department of Experimental Immunohematology, Sanquin Research and Landsteiner Laboratory, Amsterdam UMC, University of Amsterdam, Amsterdam, Netherlands

**Keywords:** Antigen binding fragment, Immunoglobulin A1, Clonal repertoires, human milk, Serum, Clonal selection, Antibodies

## Abstract

The most abundant immunoglobulin present in the human body is IgA. It has the highest concentrations at the mucosal lining and in biofluids such as milk and is the second most abundant class of antibodies in serum. We assessed the structural diversity and clonal repertoire of IgA1-containing molecular assemblies longitudinally in human serum and milk from three donors using a mass spectrometry-based approach. IgA-containing molecules purified from serum or milk were assessed by the release and subsequent analysis of their Fab fragments. Our data revealed that serum IgA1 consists of two distinct structural populations, namely monomeric IgA1 (∼80%) and dimeric joining (J-) chain coupled IgA1 (∼20%). Also, we confirmed that IgA1 in milk is present solely as secretory (S)IgA, consisting of two (∼50%), three (∼33%) or four (∼17%) IgA1 molecules assembled with a J-chain and secretory component (SC). Interestingly, the serum and milk IgA1-Fab repertoires were distinct between monomeric, and J-chain coupled dimeric IgA1. The serum dimeric J-chain coupled IgA1 repertoire contained several abundant clones also observed in the milk IgA1 repertoire. The latter repertoire had little to no overlap with the serum monomeric IgA1 repertoire. This suggests that human IgA1s have (at least) two distinct origins; one of these produces dimeric J-chain coupled IgA1 molecules, shared in human serum and milk, and another produces monomeric IgA1 ending up exclusively in serum.

## Introduction

The antibody humoral immune response is part of the adaptive immune system and responds to foreign antigen exposure, including naturally acquired infections and vaccinations, driven by B cells that produce immunoglobulins (Igs). When a new antigen is encountered, B cells respond by proliferating daughter B cells, forming antibody secreting plasma cells (ASPC) or memory B cells [1, 2]. The antigen specific ASPCs are capable of producing high numbers of the same Ig molecule, which can effectively bind, neutralize, and mark potentially harmful foreign antigens for destruction by other immune components [3]. Antibodies make up a large part of all proteins in the serum proteome (roughly 15–25%) [4–6], but they are also highly abundant in other biofluids such as human milk [7–10]. Although the humoral immune response has been intensively studied for decades, we still lack a full understanding regarding where all Ig molecules originate, such as from which specific lymph nodes and/or secondary lymphatic organs, and how they get to the sites and biofluids where they are active [11].

Regardless of their origin, all antibody classes are produced by B cells and are composed of two light chains (LC) and two heavy chains (HC). The intact antibody can be functionally divided into three fragments: two antigen-binding domains fragments (also known as fragment antigen-binding or Fab) at the N-terminal side, and the so-called a constant domain (also known as fragment crystallizable (or Fc) at the C-terminal side. The Fc can bind to Fc-receptors on immune cells and mediate immune effector responses such as phagocytosis, antibody-dependent cell-mediated cytotoxicity, respiratory burst, and cytokine release [12, 13]. While the Fc has a conserved sequence and structure, in contrast the Fab contains the variable (V) regions which are responsible for specific recognition of vastly diverse antigens and is hypervariable in sequence. A major cause of this hypervariability is that the antigen-binding domains of the LC and HC are encoded through a random assembly of one of multiple V(-D)-J gene segments that recombine to a staggering number of combinations, together encoding a variable region that is unique for each naïve B cell [14]. Natural polymorphisms, somatic hypermutation (SHM), and class switching further expand the eventual diversity in antibody repertoires [15].

Each Ig derived from a different B cell is unique, resulting in a theoretical estimate of 10^16^–10^18^ unique Ig sequences that can be made throughout the human body [16]. However, we recently showed that at the protein level in human serum and milk both IgG1 and IgA1 clonal repertoires are much simpler, dominated by only tens to hundreds of clones at a given time, albeit unique for each donor [17, 18]. In other words, a few hundred clones of IgG1/IgA1 constitute between 50–90% of all IgG1/IgA1 molecules at a given time and this is true both in human serum and milk. In the current study, we focus on IgA1 and aim to address whether there are within single donors differences or similarities between human serum and milk IgA, from both a structural point of view as well as in their IgA1 clonal repertoires.

Humans have two subclasses of IgA, IgA1 and IgA2. The IgA subclass structures are highly homologous, with the exception of the hinge region, which is elongated for IgA1 and shorter in IgA2, resembling more the hinge found in IgG1. Serum IgA is thought to be predominantly monomeric and present at concentrations ranging from 1–3 mg/mL [19, 20], while at mucosal epithelial cell surfaces IgA is mostly found in a dimeric form [21]. Dimeric IgA has been reported to be produced specifically by plasma cells in the lamina propria of mucosal epithelial cells, and these dimers consist of two monomeric IgA molecules joined together at the Fc region by the joining (J-) chain [22]. Recently, Kumar *et al*. resolved cryo-electron microscopy structures of dimeric, tetrameric, and pentameric secretory (S)IgA-Fcs interacting with the J-chain [23]. These structures support a mechanism wherein the J-chain serves as a template for Ig oligomerization in a similar fashion as seen for IgM [24]. These J-chain coupled immunoglobulins can bind to the polymeric immunoglobulin receptor (pIgR) and be transported across the epithelium into the lumen of mucosal surfaces. The pIgR is then enzymatically cleaved to form the secretory component (SC). Together, J-chain coupled IgA and SC are called SIgA [25]. Further, Bharathkar et al. resolved cryo-electron microscopy structures of dimeric murine IgA and SIgA elucidating how the interaction of SC with J-chain coupled IgA changes the overall structure of the antibody and its antigen binding interactions [26]. In human milk the concentration of SIgA has been reported to range from 0.2 to 80 mg/mL [7–10].

The different (hetero) oligomeric assemblies of IgA throughout the body perform a plethora of functions, most of which aim to balance pro- and anti-inflammatory events, maintaining homeostatic conditions [20, 22, 27–31]. Generally, mucosal IgA is considered to be anti-inflammatory, well situated with higher avidities due to its oligomeric nature, to neutralize pathogens before they have a chance to invade or cause harm. If the perimeters are breached, serum IgA and IgG with their much stronger proinflammatory properties serve as the next line of defense. The question remains whether all forms of IgA originate from the same pool of B cells.

Here, we aimed to explore and compare the clonal repertoire of serum and milk IgA to elucidate whether they originate from the same B cells. To achieve this, we analyzed IgA1 in matched serum and milk samples from several individuals and looked at both the oligomeric state and the clonal variable regions, i.e. the Fab profiles. We monitored these repertoires over time to determine the overlap (or lack thereof) within each donor and between donors and biofluids. This approach revealed clonal overlap between the dimeric J-chain coupled IgA present in both serum and milk, whereas the monomeric serum IgA1 clonal repertoire was found to be clearly distinct.

## Results

### Experimental design

**Fig. 1:**
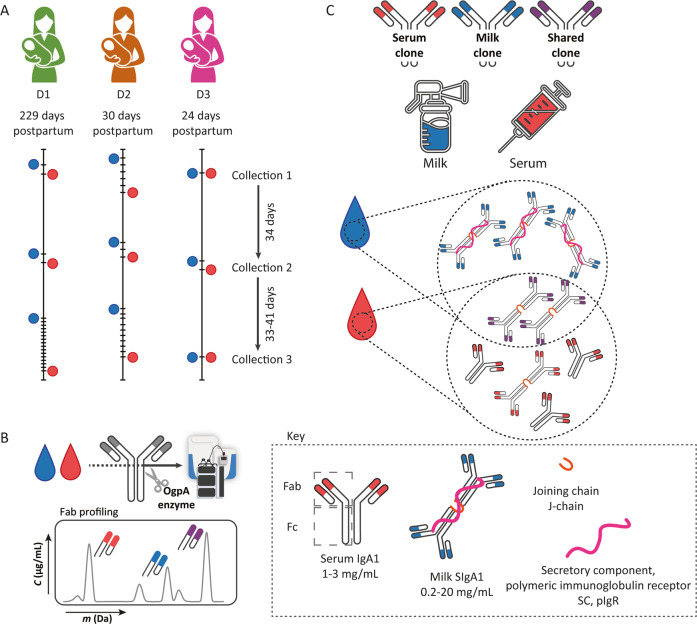
Experimental design. **A** Three individual donors with matching serum and milk samples were selected for longitudinal IgA1 Fab clonal profiling. Samples were collected at three time points spaced approximately one month apart (see Supplementary Table S1 for further details). The predominant IgA1 structures in human serum (monomer, with indicated Fab and Fc) and milk (secretory dimer, with J-chain and SC), with their expected concentration ranges, are indicated in the key at the bottom. **B** Human serum and milk were subjected to selective IgA capture, followed by IgA1 digestion (by OgpA), and LC-MS analysis of the released intact IgA1 Fab molecules to provide clonal repertoires. Each LC-MS peak with a unique mass and retention time was annotated as a unique clone. **C** From these 18 samples, we compared the IgA1 Fab clonal profiles over time within and between donors and sample types. Human serum and milk IgA1 repertoires were compared for unique and shared clones between donors and biofluids

Using a recently introduced mass spectrometry-based approach allowing for IgA1 clonal profiling from human serum and milk [18], we aimed to explore the differences and similarities between inter- and intra-donor serum IgA1 and milk SIgA1 clonal repertoires. To achieve this, serum and milk samples were analyzed from three donors that participated in a previously described cohort [32, 33]. The three donors ranged in age from 27 to 35 years at delivery (see Supplementary Table S1 for details). We selected donors for which paired serum and milk samples had been donated at three time points, each approximately one month apart (Fig. 1A). Human serum and milk were spiked with two monoclonal antibodies (mAbs) of the IgA1 isotype as internal standards, and then all IgA was purified using affinity enrichment and subsequently subjected to overnight cleavage by the *O*-glycan targeting protease OgpA. This enzyme cleaves the protein backbone N-terminally of *O*-glycan bearing amino acids, allowing for the selective cleavage of IgA1 in its hinge region, regardless of the oligomeric state [18]. This releases specifically the IgA1 Fab fragments covering the entire variable domain, removing the IgA1 Fc portion containing the *O*- and *N*-glycans and furthermore, leaving bound IgA2 unaffected. Subsequently, these released IgA1 Fab molecules (with masses ranging from typically 45 < Mw < 51 kDa) were subjected to liquid chromatography coupled mass spectrometry (LC-MS) for separation, mass analysis, annotation, and quantification of the individual intact Fab clones, allowing us to monitor the repertoires of IgA1 both qualitatively and quantitatively (Fig. 1B).

### Longitudinal clonal profiles of human serum and milk IgA1

**Fig. 2:**
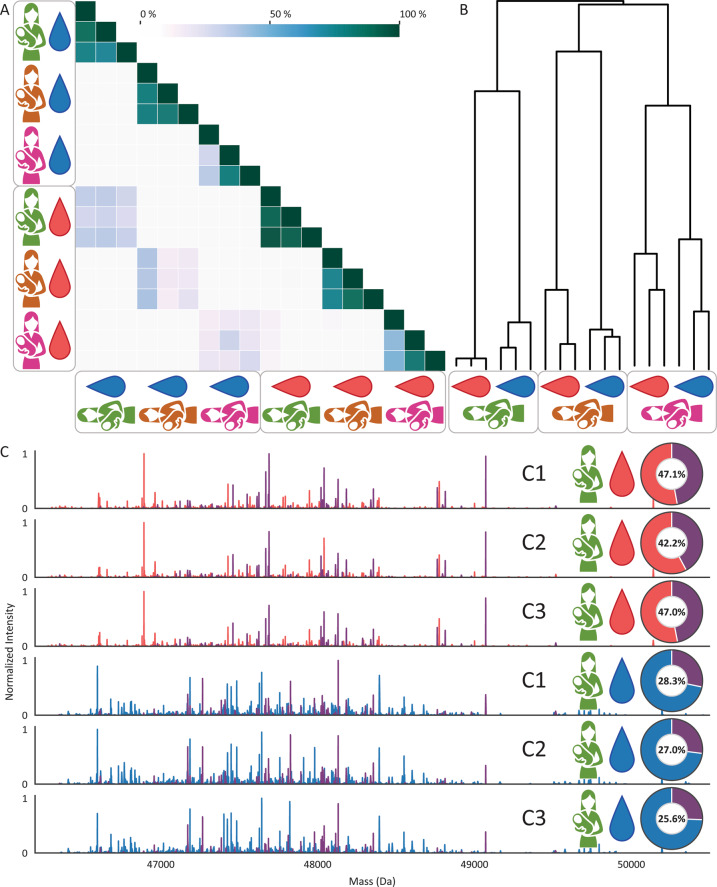
IgA1 Fab profiling reveals highly unique and stable human serum and milk intra-donor IgA1 clonal repertoires, with several clones shared between serum and milk. **A** The overlap between the full clonal IgA1 repertoires is given as a color-coded percentage of the total IgA1 clone abundances. Donors are annotated as colored silhouettes following Fig. 1. Data for serum and milk are annotated as red and blue droplets, respectively, with three rows/columns per donor group, one for each sample collection. **B** Hierarchical clustering dendrogram reveals the tight connection between IgA1 clonal repertoires from the same donor. Within a single donor the samples from either serum or milk cluster most tightly with each other. Additionally, there is close clustering between the IgA1 repertoires from serum and milk within a given donor. Samples taken from different donors are completely dissimilar. **C** Illustrative deconvoluted IgA1 Fab mass and concentration profiles, all obtained from Donor 1. Sample collections are annotated as C1–3, and serum and milk are indicated by red and blue droplets, respectively. Each peak represents a unique Fab (based on unique retention time and mass combination) and the peak height depicts the abundance of that clone. Red and blue peaks represent clones uniquely identified in either serum or milk, respectively. The purple peaks represent clones detected in both serum and milk. On the right of the mass profiles, the pie charts depict the percentage (in concentration) of shared clones in each measured repertoire. The red and blue pie slice represents the combined relative concentrations of clones uniquely identified in either serum or milk, respectively. The purple pie slice represents the combined relative concentration of shared clones, with the number inside the chart depicting the percentage of shared clones

To investigate the clonal diversity and possible similarity of IgA1 clones in serum and milk, we incubated purified serum and milk IgA samples with OgpA and subjected the released Fab fragments to intact LC-MS analysis. We found that the longitudinal clonal profiles had a high percent overlap within one donor, particularly within the same biofluid (Fig. 2A). The overlap between IgA1 repertoires of different donors was close to zero. Notably, the comparison of matched serum and milk samples revealed substantial intra-donor clonal overlap between the IgA1 repertoires in serum and milk. These data stand out in the heatmap as they form a second colored diagonal throughout (Fig. 2A). High correlation between serum and milk of the same donor was confirmed by using hierarchical clustering of the clonal repertoires (Fig. 2B). This analysis showed that different collections from the same donor and same biofluid cluster most tightly together, but different biofluids within a donor also cluster together. Again, nearly no correlation was observed between different donors.

To visualize the overlap between biofluids, the complete Fab mass profiles of Donor 1 are depicted in Fig. 2C for the three serum samples and the three milk samples. In these mass profiles, each mass peak is colored according to the sample in which it was detected; red and blue peaks are exclusively detected in serum or milk, respectively. The intensity of the peaks directly reflects their relative concentration. The purple peaks depict clones that are detected in both serum and milk. In total, 379–397 IgA1 clones were identified in these serum samples, while slightly more SIgA1 clones (585–607) were identified in the human milk samples. Of all these clones, roughly 100 were abundant in both serum and milk. Comparing the repertoires based on abundance shows that ∼45% of the total IgA1 clonal repertoire detected in serum was also detected in milk, whereas the shared clones represent ∼27% of the milk IgA1 repertoire. This data reveals that within a single donor a sizable part of the IgA1 clonal repertoires of human serum and milk are shared between these two biofluids.

### Validation of shared clonality by top-down fragmentation of selected clones

**Fig. 3:**
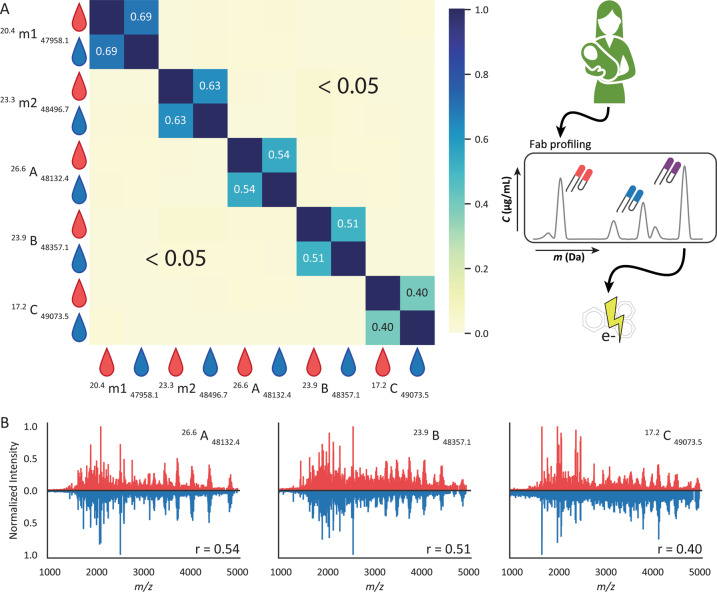
Shared clone identity in serum and milk confirmed by top-down ETD-based MS/MS analyses. **A** clones m1 and m2 refer to the spiked-in mAb standards, and clones (**A**, **B**, and **C**) (annotated as ^[RT]^ [clone] _[mass]_) were selected as shared clones between serum and milk of Donor 1. Data for serum and milk are shown as red and blue droplets, respectively. Each square in the heatmap represents a Pearson correlation value between 0 and 1 of the raw top-down ETD MS/MS spectra of the two Fab clones. **B** Mirrored top-down ETD MS/MS spectra of clones (**A**–**C**) in serum (red) and milk (blue). For each of the selected clones the fragmentation spectra in serum and milk are compared, resulting in the Pearson correlation value (r) given. See Supplementary Fig. 1 for illustrative top-down ETD MS/MS data of non-paired clones, displaying correlations below 0.05

Our approach allows for the assignment of shared clones in different samples based on their unique Fab mass and retention time (RT), annotated as ^[RT]^ [clone] _[mass]_. Nevertheless, we aimed for additional evidence to confirm that the shared Fabs between serum and milk are indeed identical. To this end, top-down MS/MS analysis using ETD fragmentation was performed on several abundant shared Fab molecules. As a control, first, the raw top-down data for the two spiked-in mAbs (^20.4^ m1 _47958.1_ and ^23.3^ m2 _48496.7_) were compared. When spiked into serum and milk these two mAbs showed quite good Pearson correlation (r) values of 0.69 and 0.63 in their raw ETD MS/MS spectra, respectively (Fig. 3A), providing reference values for identifying the same antibody in different samples. Agreeably, the correlation between the top-down data of overlapping serum and milk clones was also found to be high, albeit somewhat lower at 0.54, 0.51, and 0.40, respectively. This reduced correlation can likely be attributed to the fact that the recombinant mAbs were present in equal abundance in serum and milk, while the concentration of the endogenous clones varied substantially between serum and milk. Correlation between MS/MS spectra of different clones was much less, typically even below 0.05 (Supplementary Fig. 1). Based on this top-down proteomics data we further confirm that paired serum and milk Fab clones with identical mass and retention time share the same sequence.

### The molecular composition of IgA assemblies in serum and milk

**Fig. 4:**
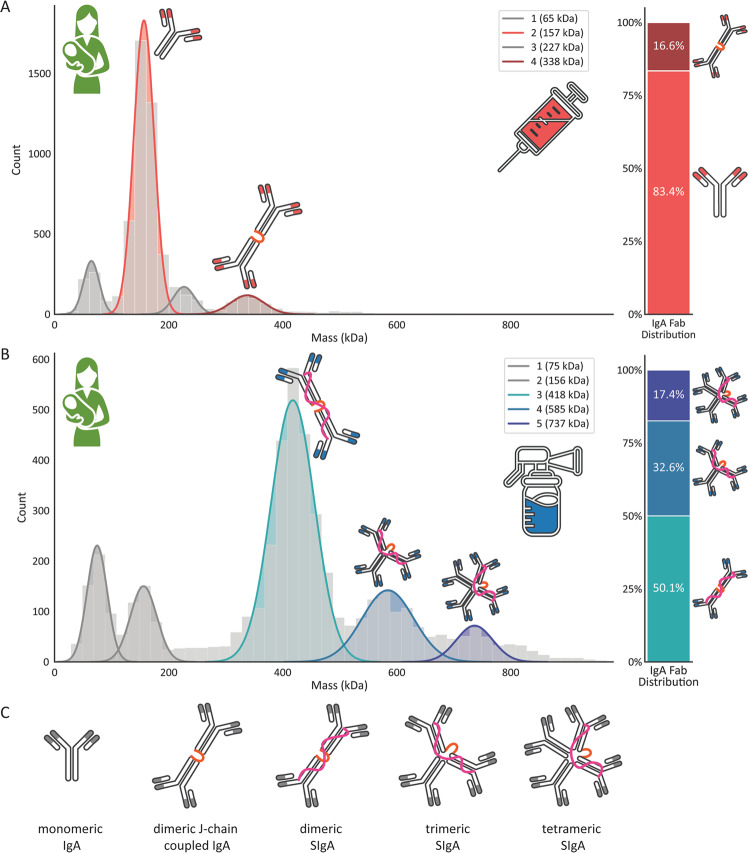
Several distinct IgA assemblies co-occur in human serum and milk as determined by mass photometry. Mass distribution histograms of IgA assemblies purified from serum (**A**) or milk (**B**). Normal distributions were fitted to the histograms depicting counts per mass bin (grey bars with a bin size of 15 kDa). From the fitted normal distributions an average mass was determined for each peak as shown in the legend. For each of the colored peaks a mass and probable composition could be assigned. A table comparing the measured masses with theoretical masses of the different IgA complexes is provided in Supplementary Tables S2 and S3. On the right adjoining the mass histograms are stacked bar charts depicting the relative contribution of distinct IgA assemblies to the total IgA concentration derived from the mass histograms, considering that dimeric J-chain coupled IgA contains twice as many Fab molecules compared to monomeric IgA, etc. **C** Depiction and description of all probable co-occurring IgA assemblies in human serum (first two structures) and milk (last three structures). Distinctively in serum, about 83% of IgA is present as a monomer, whereas 17% is present as a dimeric J-chain coupled IgA. In milk a distribution of SIgA assemblies is detected, all containing one J-chain, one SC, and two to four IgA molecules. The grey peaks are likely co-purified albumin and unknown (in **A**, 65 and 227 kDa, respectively), and possibly free SC partially degraded by the acid treatment in purification (in **B**)

Shared clones in serum and milk should originate from the same B cell. The presence of a clone in milk requires the co-expression of a J-chain for binding to pIgR and further transportation into milk. Therefore, we hypothesized that the same clone detected in human serum and milk should be present as dimeric J-chain coupled IgA in serum. Therefore, we next set out to explore the molecular composition of total IgA in human serum and milk, as current literature is not entirely consistent in this respect. Total IgA was captured from serum or milk from Donor 1 at collection 1 (C1), and subjected to mass analysis by mass photometry (MP) to assess the oligomeric state. MP is a label-free light scattering-based mass analysis technique, which allows for the determination of the mass of individual particles [34–36]. The resulting mass distribution histograms of purified serum and milk IgA are depicted in Fig. 4A, B, respectively. Both mass histograms show a variety of co-occurring molecular assemblies with masses roughly between 150 and 800 kDa.

In the IgA purified from serum (Fig. 4A), the mass distribution histogram showed a dominant peak at ∼157 kDa, which is the molecular mass of monomeric IgA. The highest molecular weight (MW) ∼338 kDa peak is assigned to dimeric J-chain coupled IgA (expected mass 341–351 kDa). The other non-annotated peaks are likely related to co-purified albumin (grey distributions). When quantifying the two peaks containing IgA molecules, J-chain coupled IgA contains two IgA molecules, we find that 83% of all IgA in serum is present in a monomeric form, with ∼17% present as dimeric J-chain coupled IgA. The mass photometry of IgA purified from human milk (Fig. 4B) is quite different and reveals a major peak at ∼418 kDa, indicative of a SIgA dimer, which includes the J-chain and SC (expected mass 414–425 kDa). This contributes nearly 50% of the IgA in milk. The other peaks in the human milk sample approach the masses of probable secretory IgA trimer at 590 kDa (∼33% of IgA Fab molecules) and secretory IgA tetramer at 770 kDa (∼17% of IgA Fab molecules). The grey distribution lower masses in Fig. 4B are possibly due to partial acid-induced dissociation of the secretory complex. Of note, SIgA from more native preparations did not show these lower mass peaks (data not shown), supporting our annotations. Of note, in contrast to the clonal profiling data, these mass photometry analyses were performed on the total IgA (sum of IgA1 and IgA2). However, bottom-up proteomics data revealed that the total amount of IgA2 was between 5 and 50 times less than IgA1 in the blood and milk of all donors (see Supplementary Table S4), and therefore IgA2 can only contribute for a minor amount to the higher order tri- and tetrameric assemblies we observe by mass photometry.

### Serum contains two distinct repertoires of IgA1

**Fig. 5:**
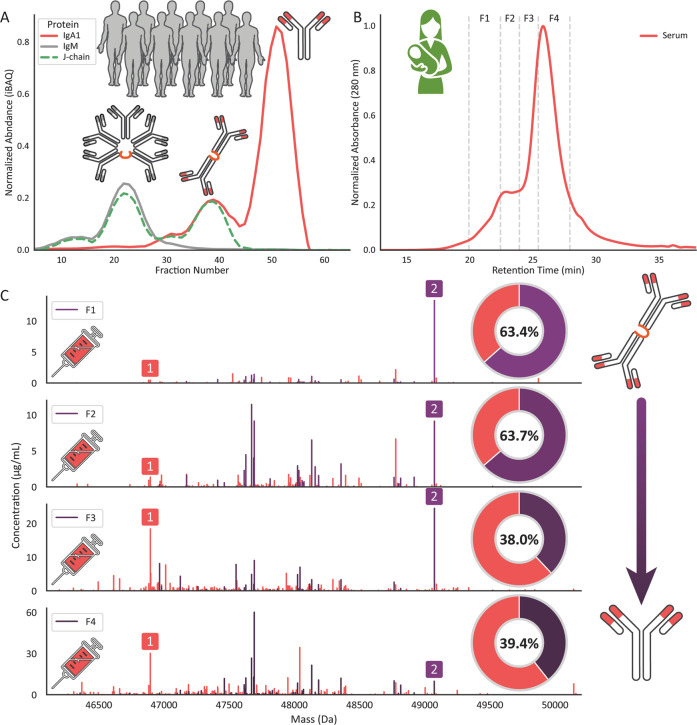
SEC fractionation confirms the presence of two distinct pools of serum IgA1 clones. **A** SEC fractionation of pooled serum from healthy donors, where each fraction was analyzed for its protein content by bottom-up proteomics. The horizontal axis depicts each analyzed fraction, and the *y*-axis depicts the normalized abundance of the proteins. IgA1 (red trace) has two peaks in the SEC chromatogram. The first, higher MW, peak coincides with the elution profile of J-chain, indicating that the first peak contains dimeric J-chain coupled IgA1. The second IgA1 peak does not contain J-chain, is of lower MW, and contains monomeric IgA1, as indicated by the cartoons. The J-chain SEC profile also has an additional peak. The higher MW peak corresponds with the elution of IgM, the other serum Ig complex that contains J-chain. **B** SEC fractionation of IgA captured from the serum of Donor 1 (C1). Fractions F1 – F4 were collected across the chromatogram, indicated by the grey dashed lines. As observed in panel (**A**), the serum profile contains two peaks corresponding to dimeric J-chain coupled IgA1 and monomeric IgA, in similar ratios as observed in (**A**). These ratios are similar to the ratios measured by MP (Fig. 4). **C** Deconvoluted IgA1 Fab mass profiles of the four serum fractions from (**B**). Each peak represents a unique Fab (based on unique RT/mass pair) and the height of each peak represents the concentration of that clone. Red peaks represent clones unique to serum, while purple peaks indicate clones shared between serum and milk. On the right of the mass profiles, pie charts are shown depicting the percentage of unique shared clones in each repertoire. In the mass profile, two peaks are highlighted by red and purple numbers

In the previous sections we showed substantial overlap between human serum and milk IgA1 repertoires (Figs. 2, 3), suggesting that this would require dimeric J-chain coupled IgA in serum. Above we demonstrated the presence of this molecular structure in serum accounting for approximately 20% of the IgA concentration (Fig. 4A). To assess whether the dimeric IgA in serum is directly related to the SIgA in milk, and likewise whether the monomeric IgA is not related, we next performed size exclusion chromatography (SEC) fractionation for Donor 1 (C1) serum and milk and in parallel on independent pooled control serum and milk samples. The control serum sample was from a pool of 10 healthy donors and the control milk sample was from one individual donor over 5 time points. For both the control serum and milk samples, shot-gun proteomics was performed on each of the SEC fractions. From Donor 1 (C1) Fab profiling experiments on the SEC fractions were performed and compared to the control experiments (Fig. 5 and Supplementary Fig. S2).

The control serum sample, pooled from several donors, was fractionated by SEC into 30 fractions, and the protein content of each fraction was analyzed by shot-gun proteomics. Proteins were quantified by label-free quantification (LFQ) to obtain a relative abundance in each fraction. Through this analysis we found that serum IgA1 displayed two distinct peaks in SEC and as expected the higher MW peak overlaps with the elution profile of the J-chain (Fig. 5A). Thus, it is possible to distinguish and fractionate two distinct pools of serum IgA1, the monomeric IgA1 pool and the dimeric J-chain coupled IgA1 pool. Furthermore, the control pooled serum sample also shows a ∼80:20 ratio of monomeric and dimeric IgA1, in agreement with the MP data for Donor 1 (C1). Notably, a second higher MW peak is observed in the elution profile of the J-chain, which coincides with the elution profile of IgM, the other Ig complex in serum that incorporates a J-chain.

Subsequently, we used this SEC fractionation approach to fractionate and analyze the purified serum IgA from Donor 1 (C1) (Fig. 5B). As expected, two distinctive maxima were observed. From Donor (C1) just four fractions (F1–F4) were collected covering the full SEC elution profile of IgA. From these four fractions, in parallel, IgA was extracted and digested to release selectively the IgA1 Fab fragments. The resulting IgA1 repertoires of these four fractions were profiled separately by LC-MS.

In the resulting IgA1 Fab mass profiles across the four serum fractions, a difference in total concentration and the concentration of individual clones was clearly observed (Fig. 5C). In the higher-MW IgA1-containing fractions (F1 and F2) the percentage of identified clones shared with the milk IgA1 repertoire is substantially higher (∼63%) than the percentage of shared clones in the later fractions (∼39%). In the mass profiles, two individual clones are highlighted and numbered. The first peak highlights a clone (1) that is present predominantly in the later fractions (F3–F4) and is therefore considered to be originating from monomeric IgA1. As the coloring of this peak indicates, it is uniquely detected in serum. Conversely, the second highlighted clone (2) is shared between serum and milk and is the most dominant peak in the earliest fraction (F1). It is far less dominant in the last fraction, and it is therefore assumed that this clone originates from serum dimeric J-chain coupled IgA1. The data substantiate our hypothesis that serum clones of the dimeric J-chain coupled pool of IgA1 are preferably shared with milk while monomeric IgA1 serum clones are less likely to be shared with milk.

SEC experiments with a milk sample from Donor 1 (C1) and in parallel a control milk sample from one donor across several time points were also performed. With the LFQ abundances from shot-gun proteomics we can distinguish between IgA1, IgA2, IgM, J-chain and SC in the SEC fractions of the control sample (Supplementary Fig. 2). Although we showed in Fig. 4 that several IgA assemblies co-exist, they cannot be clearly distinguished from SEC data (from Donor (C1) data not shown). However, the data from the control milk samples does confirm that there is little to no monomeric IgA present in milk since all IgA signal correlates with J-chain, which indicates that the low MW species observed in Fig. 4 are not from monomeric IgA. The high abundance of free SC in the control milk sample is not surprising as this is commonly reported in human milk literature [37–39].

## Discussion

The human body produces a vast amount of IgA molecules, reaching up to 60 mg per kg of body mass per day [21, 40, 41]. Produced by dedicated B cells, these molecules end up in the circulation and form an important part of the human immune response. While IgA is distributed throughout the body, it is predominantly found at mucosal surfaces and in biofluids like blood, saliva, and milk. Here we observed and evaluated (dis)similarities between human serum and milk IgA, both from a structural point of view as well as in their clonal repertoires. We discovered exciting new features regarding the immune biology of IgA1. A considerable part of the IgA1 clonal repertoire is shared between serum and milk within a donor. This shared pool of IgA1 is structurally specific, containing a J-chain in their assembly and consisting of dimeric molecular assemblies.

The personalized longitudinal IgA1 Fab clonal profiling of human serum and milk from individual donors revealed that the intra-donor IgA1 clonal profiles are considerably stable, with a high degree of correlation, over time within a single biofluid. However, no measurable inter-donor correlations were observed. As individuals develop unique acquired immunity throughout their lifetimes due to different genetics and environmental exposures, it may come as no surprise that IgA1 clonal repertories are distinct between individual donors. This is also in line with our previous findings for human serum IgG1 and milk IgA1, wherein no overlap between clonal repertories was observed between individuals [17, 18]. Also not unexpectedly, we found that both biofluids harbored a substantial fraction of unique clonal repertoires for either serum or milk. Surprisingly, given these fractions of unique repertoires within a donor there was considerable overlap for the clonal repertoires of IgA1 found in serum and milk. Furthermore, we show that the overlap between the clonal repertoires of IgA1 in serum and milk arises chiefly from the dimeric J-chain coupled IgA1, and not from other molecular forms of IgA1 present in either biofluid.

Understanding the structural assemblies of IgA may provide insights into the B cell origins of these antibodies and even their function. It is often assumed that human milk IgA is mostly dimeric, albeit that trimeric and tetrameric forms of J-chain coupled IgA have been reported already 25 years ago [41]. By utilizing multiple mass-based analyses, MP and SEC-LC-MS, we revealed that in our human milk samples, IgA is present solely as SIgA, in a molecular composition of (IgA)_*n*_ J-chain_1_SC_1_, with 2 < *n* < 4, with ∼50% being dimeric, 33% trimeric and 17% tetrameric. Finally, we show that in human serum IgA is present in the form of “bare” monomers as well as J-chain coupled dimers in a ratio of approximately 80:20. The presence of IgA1 dimers in serum has been reported [21, 42, 43], but is here solidly confirmed with modern techniques. In particular, the clear presence of the J-chain and the relatively high abundance of the dimer complex are convincingly demonstrated by our SEC-LC-MS data. These results should increase the awareness that higher-order molecular assemblies of IgA exist as dimers in blood. Likewise, a considerable fraction of secretory IgA exists as trimers and tetramers. Although we here focus on IgA1—being by far the more abundant IgA subclass in the samples we analyzed (see Supplementary Table 4)— we expect that also IgA2 will contribute to these multimeric molecules in serum and milk. Their presence is interesting as multimeric forms of IgA may affect both protective capacity in infectious diseases and pathogenic potential in autoimmune diseases [21, 27].

**Fig. 6:**
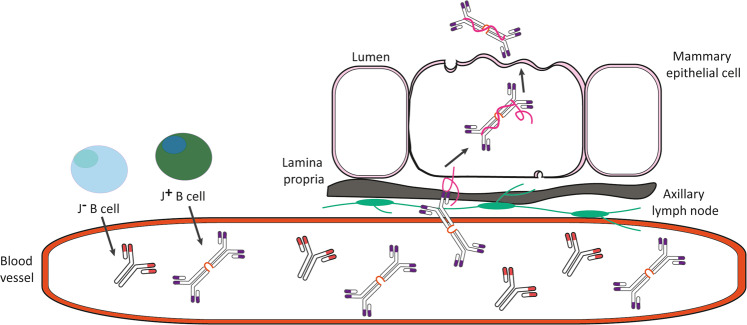
Model based on the co-occurrence of J^+^ or J^−^ B cells leading to structurally different IgA1 assemblies in serum and milk. A blood vessel is depicted with antibodies originating from either J^+^ or J^**−**^ B cells, which expressing either dimeric J-chain coupled IgA1 or monomeric IgA1, respectively. The representation of each J^+^ or J^**−**^ B cell is depicted in the dashed box in the top left corner. Only the J^+^ B cells expressing dimeric J-chain coupled IgA1s are shared between human serum and milk. These shared IgA1s can enter the serum or mammary epithelial cells by axillary lymph nodes and be picked up by pIgR to be transported from the lamina propria to the lumen where it is expressed as SIgA1

To better understand where the structural and clonal similarity and diversity between human serum and milk IgA1 originate from, it is important to address how and where these molecules are formed, and how they end up ultimately in serum and/or milk of an individual. Like all human antibodies, IgA1 is produced by B cells. A review by Castro and Flajnik provides a historical overview of the literature regarding IgA producing B cells [44]. The accumulated data from several experiments performed in different species (e.g., humans, mice, sharks) results in a complicated, sometimes contradicting, picture. The general view is that all B cells derive from either B1 or B2 lineages to form a rearranged B-cell receptor. These eventually give rise to their secretion as soluble and class switch Ig-molecules (including IgA1) after recognition of their cognate antigen, proliferation and differentiation into plasma cells, which can co-express J-chain or not, termed J^**+**^ or J^**−**^ B cells. The J^**−**^ B cells (the B1b or B2 cell lineages), would then be responsible for producing monomeric “bare” IgA1, whereas those that express the J-chain (B1a lineage) would lead to dimeric IgA secreting cells. It has also been stated that B2 cell-derived monomeric IgA is dominant in serum and has a different V gene repertoire than those originating from the B1 lineage [44, 45]. In Fig. 6 we incorporated how our data relates to this model, where there are J^**+**^ or J^**−**^ B cells, which express either dimeric J-chain coupled IgA1 or monomeric IgA1 respectively, and only the J^**+**^ B cells expressing dimeric J-chain coupled IgA1 are shared between human serum and milk. Although the picture is far from well-defined, there are also multiple B cell lineages producing IgA1 molecules of different composition and structures in humans. Moreover, these distinct B lineages also likely lead to IgA1 molecules with different functional properties, that can balance pro- and anti-inflammatory events maintaining homeostatic conditions [22, 27]. Potentially then homeostatic pro- and anti-inflammatory conditions could be therapeutically stimulated by increasing the production of either B1 or B2 lineages. However, more needs to be known to fully understand the mechanisms of IgA1 mediated effector functions.

The data we present here on the structural and clonal properties of IgA1 provide more clarity to these previous reports. They confirm quite a few of the older findings and partially explain why we find both unique as well as overlapping IgA1 clones in serum and milk from a single donor. From this we conclude that the unique clones observed only in the serum, and not in milk, originate from monomeric “bare” IgA1. Clones found in both serum and milk from a single donor, originate from J-chain containing IgA1 complexes and are SIgA1 in milk and dimeric J-chain coupled IgA1 in serum. Together indicating that the shared dimeric J-chain coupled IgA1s in human serum and milk likely originate from the same J-chain producing B cell. This reasoning is supported by a recent publication wherein Iversen *et al*. showed clonal relatedness between blood and gut biopsies of celiac disease patients [46]. They suggested that an antigen response at the intestinal epithelium was responsible for the shared IgA clones between the gut and serum. Based on our findings it is likely that indeed a tissue-specific J^+^ B cell produces dimeric J-chain coupled IgA that ends up as both SIgA and circulating dimeric IgA. It will be interesting to see whether clonal relatedness extends beyond a single secretory production site like the gut or if indeed terminal differentiation occurs locally in some form of secondary lymphoid tissue, as for example suggested by Brandtzaeg et al. [47].

Now that we have shown that serum contains two very distinct repertoires of IgA1 clones, the obvious next question that needs to be addressed is whether these two repertoires are functionally distinct. Here, we may ask questions such as whether reported IgA responses to pathogens and or vaccinations are due to either monomeric or J-chain coupled IgA1. A relevant example is the reported IgA response to SARS-CoV-2 infection or vaccination. We mention this example as recently it has been proposed that IgA may dominate the early neutralizing antibody response to SARS-CoV-2 [48], and that dimeric IgA may result in better SARS-CoV-2 neutralization [49]. In principle, with the right samples being available the presented MS-based approach could address such important immunological questions for SARS-CoV-2, but also for many other infections or diseases involving an IgA immune response.

## Materials and methods

### Donor and study population

All samples used in this study are a subset from a larger longitudinal follow-up study of the COVID MILK study [32, 33]. The Medical Ethics Committee of Amsterdam UMC approved the study. Written informed consent was obtained from all participants before participation. Data was collected one and two months after the initial sample collection. From these 24 women we selected 3 donors here for longitudinal IgA Fab clonal profiling, all serum and milk samples were collected over three sampling points over an extended period. For all three time points, participants were requested to collect 100 mL of human milk in specially provided bottles and instructed to store the bottle in their home freezer until collected by study staff during a home visit. Subsequently, the samples were transported back to the lab where they were stored at −80 °C until analysis. During the home visits, maternal serum was collected by a trained phlebotomist.

The pooled human serum, used as a control sample, was obtained from healthy volunteers as previously described [50, 51]. The control human milk samples, collected from one donor across 5 time points have been previously described [52]. Recombinant monomeric IgA1 targeting CD20 (7D8-IgA1) or MET (5D5v2-IgA1), used as internal standard, allowing quantification, was an in-kind gift from Genmab (Utrecht, NL).

### IgA enrichment, capture and digestion

The methods for IgA Fab profiling have previously been extensively described [18]. Briefly, all IgA was captured using CaptureSelect IgA affinity matrix (ThermoFisher Scientific). The bead slurry was added to Pierce spin columns with screw cap (ThermoFisher Scientific) and repeatedly washed with PBS. Following the washing steps, samples were added to the spin columns with a plug inserted into the bottom of each spin column. For either human milk or serum samples, an expected IgA quantity of 40 µg was added to equivalent bead slurry. PBS and 200 ng of both IgA1 mAbs (7D8-IgA1, 5D5v2-IgA1), as spike-in standard, were added to the samples. Samples were then incubated for 1 h while shaking at 750 rpm at room temperature in and Eppendorf thermal shaker (Eppendorf, The Netherlands). Following the incubation, the plugs were removed from the spin columns and the flowthrough was collected by centrifugation. Then the beads with captured IgA were washed four times with PBS.

For the hinge region digestion of IgA1 we relied on the O-glycopeptidase from Akkermansia muciniphila, OgpA (OpeRATOR®, Genovis, Llund, Sweden). Digestion was performed by adding 50 µL PBS containing 40 U SialEXO (a sialidase cocktail to remove sialic acids from the O-glycans) and incubating for 1 h at 37 °C with continuous shaking at 750 rpm. Then 1 µL (40 U) of OgpA enzyme was added, and incubation was continued overnight, in an Eppendorf thermal shaker (Eppendorf, The Netherlands). Following overnight digestion with OgpA, 20 µL of pre-washed Ni-NTA agarose slurry (1:1 in PBS) was added to the spin columns and incubated for an additional 30 min. Then the plug was removed from the column, and the flowthrough, containing the IgA1 Fabs, was collected by centrifugation.

### Fab profiling by LC-MS and LC-MS/MS

The LC-MS and data processing approaches as previously described by Bondt *et al*. were applied [17, 18]. In short, the collected Fab samples were separated by reversed phase liquid chromatography on a Thermo Scientific Vanquish Flex UHPLC instrument, equipped with a 1 mm × 150 mm MAbPac reversed phase analytical column, maintained at 80 °C during chromatographic separation. The LC was directly coupled to an Orbitrap Fusion Lumos Tribrid mass spectrometer (Thermo Fisher Scientific, San Jose, California, USA). Fab samples were injected as 10 µL and subsequently separated over a 62 min gradient at a flow rate of 150 µL/min. Gradient elution was achieved using mobile phases A (0.1% HCOOH in Milli-Q HOH) and B (0.1% HCOOH in CH_3_CN), see previous publications for gradient details [17, 18]. The instrument was operating in Intact Protein and Low-Pressure mode for the acquisition of MS data. Spectra were recorded with a resolution setting of 7500 (@ 200 m/z) in MS1. Scans were acquired in the range of 500–4000 m/z with an AGC target of 250% and a maximum injection time set to 50 ms. For each scan 5 µscans were recorded.

For LC-MS/MS acquisition the same LC setup and gradient were employed. MS/MS data were only collected for Donor 1. MS1 scans were acquired from 500–3500 m/z at a resolution of 7500 (at 200 m/z) with an AGC target of 250% and a maximum injection time of 50 ms. For each scan 2 µscans were recorded. MS/MS scans were acquired using a data-dependent acquisition (DDA) using top 1 selection. Peaks were selected with a 4 *m/z* isolation window, electron transfer induced dissociation (ETD) was achieved with a 16 ms reaction time with a 1e6 ETD reagent target. Scans were recorded with a 350–5000 m/z mass range, at a resolution of 120,000 (at 200 *m/z*) with an AGC target of 10,000% and a maximum injection time set to 500 ms. For each scan 5 µscans were recorded.

### Mass photometry

Mass photometry experiments were performed to determine the co-occurrence of (hetero) oligomeric IgA structures in human milk and serum. Approximately 40 µg of IgAs were captured from either human milk or serum using CaptureSelect IgA affinity matrix. After capturing, IgAs were washed three times with PBS followed by elution with 0.1 M glycine-HCl (pH 2.7) and neutralized with 1 M Tris (pH 8.0). The respective human milk and serum IgAs were then diluted with PBS (between 5 and 15 times until sufficient signal was obtained) and measured using a mass photometer (Refeyn, Oxford UK). Prior to the measurement, the borosilicate microscope coverslips (24 × 50 mm 1.5H, Marienfeld) were cleaned in four sequential sonication rounds of 5 min using isopropanol and Milli-Q (2×). Silicone cell culture gaskets (50 wells, 3 mm diameter × 1 mm depth, Grace Bio-Labs) were cut into sets of four wells and placed onto a clean coverslip. Then 12 µL PBS was added to the coverslip wells to focus the instrument on the glass-liquid interface. For data acquisition, all samples were measured in triplicate by adding 3 µL of diluted sample directly into the PBS-loaded well to a final concentration of 1–30 nM, followed by recording for 150 s (12,000 frames). Recordings were processed in DiscoverMP (Refeyn, Oxford UK) and calibrated using an in-house calibration mix consisting of proteins with previously determined accurate masses by MS (73 kDa IgG4Δhinge-L368A, 149 kDa IgG1-Campath, 483 kDa apoferritin, and 800 kDa E. Coli GroEL).

### IgA fab preparation from size exclusion chromatography (SEC)

To investigate whether human milk and serum had distinct and/or overlapping IgA Fab clones SEC was performed to fractionate the different IgA1 containing complexes. From Donor 1, 400 µg IgA from time-matched human milk and serum samples (from collection 1) were affinity captured. Samples were mixed with CaptureSelect IgA affinity matrix and mixed head over head at RT for 2 h on an Eppendorf ThermoMixer C equipped with SmartBlock 1.5 mL tabletop rotator. The total volume of the sample mixture was brought up to 3 mL in PBS for mixing. After mixing, samples were passed over spin columns in 500 µL increments and then washed two times with PBS (500 µL) and two times with Milli-Q water (500 µL). After washing, IgAs were eluted with two times 300 µL 0.1 M glycine-HCl (pH 2.7). The collection tube contained 120 µL of 1 M Tris (pH 8.0) to neutralize the eluted sample. After each addition of glycine, the samples were mixed at 750 rpm at RT for 10 min followed by centrifugation at 500 × *g* for 1 min RT.

The captured IgAs (~600 µL) were concentrated by passing the samples over 30 kDa filters (Vivaspin, Sartorius Stedim Biotech). Approximately 100 µL of sample was passed through the filter by centrifugation at 10,000 × *g*, for 3.5 min at 4 °C until a final volume of 120 µL remained. The concentrated IgAs were then fractionated on SEC, as described below.

The collected fractions were then pooled by the expected oligomeric state for both human milk and serum and again IgAs were captured on beads with a 1% blocking buffer background (Bio-Rad, Netherlands) and addition of the 7D8-IgA1 and 5D5v2-IgA1 at 0.2 µg/mL. The samples were then mixed head over head for two hours at RT. After mixing, the samples were passed over spin columns in 500 µL increments to collect all captured IgAs for overnight digestion. From this point forward, the standard protocol as described above was followed, including LC-MS analysis of the released IgA1 Fabs.

### SEC separation of human serum and milk samples

For SEC experiments either purified IgAs from human serum and milk, pooled human serum or longitudinal human milk samples were analyzed with the same method. An Agilent 1290 Infinity HPLC system (Agilent Technologies, Waldbronn. Germany) consisting of a vacuum degasser, refrigerated autosampler with a 100 µL injector loop, binary pump, thermostated two-column compartment, auto collection fraction module, and multi-wavelength detector, was used in this study. The dual-column set-up, comprising a tandem YarraTM 4000-YarraTM 3000 (SEC-4000, 300 × 7.8 mm i.d., 3 µm, 500 Å; SEC-3000, 300 × 7.8 mm i.d., 3 µm, 290 Å, Phenomenex, Netherlands) two-stage set-up was used. The columns were cooled to 17 °C while the other bays were chilled to 4 °C to minimize sample degradation. The mobile phase buffer consisted of 150 mM ammonium acetate in water and 50 mM Arginine. For purified IgAs, 40 µL were injected per sample three times. For the fresh control serum, approximately 1.25 mg of protein was injected per run. The proteins were eluted using isocratic flow within 60 min, and the flow rate was set to 500 µL/min. In total, 74 fractions were collected within a 20–42 min time window using an automated fraction collector. For purified IgAs, multiple injections were collected into the same consecutive wells of the fraction collector. The chromatograms were monitored at 280 nm.

### LC-MS/MS analysis of SEC fractions of control serum and milk samples

Bottom-up LC-MS/MS analysis was used to determine the SEC elution profile of all proteins in a control pooled human serum sample and five control human milk samples from a single donor. Both serum and milk samples were treated under the same conditions. The 74 collected SEC fractions were digested by trypsin, and analyzed by a bottom-up proteomics, as described previously [53]. Briefly, the separation of digested protein samples was performed on an Agilent 1290 Infinity HPLC system (Agilent Technologies, Waldbronn, Germany) coupled to a Q Exactive™ HF (Thermo Scientific, Bremen, Germany). Samples were loaded on a 100 µm × 20 mm trap column (in-house packed with ReproSil-Pur C18-AQ, 3 µm) (Dr. Maisch GmbH, Ammerbuch-Entringen, Germany) coupled to a 50 µm × 500 mm analytical column (in-house packed with Poroshell 120 EC-C18, 2.7 µm) (Agilent Technologies, Amstelveen, The Netherlands). From each SEC fraction, 10 µL of digest was injected, with a run time of 60 min and flow rate of 300 nL/min. The mass spectrometer was operated in positive ion and data-dependent acquisition mode. Full MS scans were acquired with 60,000 resolution (@ 200 m/z), a scan mass range of 375 to 1600 m/z and automatic Gain Control (AGC) target of 3e6 with a maximum injection time of 20 ms. Full scan MS/MS was acquired at 30,000 resolution (@ 200 m/z), with a mass range of 200 to 2000 m/z and an AGC target of 1e5 with a maximum injection time defined at 50 ms. 1 µscan was acquired in both full MS and MS/MS scans. The LC-MS/MS data were searched against UniProtKB/Swiss-Prot human proteome sequence database with MaxQuant software. The intensity of each detected protein in each of the 74 subsequent SEC fraction was determined by using iBAQ values, to obtain detailed SEC elution profiles of each protein. We used the obtained data for IgA1, IgA2, IgM pIgR and the J-chain protein for comparative analysis to samples in the current study.

### Bottom-up proteomics

Methods for serum and milk sample preparation for bottom-up proteomics analysis have been extensively detailed in previous studies [54–56], as well as details on the data analysis [56].

### IgA1 clonal profiling data analysis

Intact masses of the eluted IgA1 Fabs were retrieved from the generated RAW files using BioPharmaFinder 3.2 (Thermo Scientific). Deconvolution was performed using the ReSpect algorithm between 5 and 57 min using 0.1 min sliding windows with a 25% offset and a merge tolerance of 30 ppm, and noise rejection set at 95%. The output mass range was set from 10,000 to 100,000 with a target mass of 48,000 and mass tolerance 30 ppm. Charge states between 10 and 60 were included, and the Intact Protein peak model was selected. Further data analysis was performed using Python 3.8.10 (with libraries: Pandas 1.2.3 [57], Numpy 1.20.3 [58], Scipy 1.6.2 [59], Matplotlib 3.3.4 [60] and Seaborn 0.11.1). Masses of the BioPharmaFinder identifications (components) were recalculated using an intensity weighted mean considering only the most intense peaks comprising 90% of the total intensity. Using the 7D8-IgA1 and 5D5v2 standards, the intensity was normalized, a relative mass shift was applied to minimize the mass error and a retention time shift was applied to minimize deviation between runs.

Components between 45,000 and 53,000 kDa with the most intense charge state above *m/z* 1000 and score of at least 40 were considered Fab portions of IgA1 clones. The clones were matched between runs using average linkage (unweighted pair group method with arithmetic mean UPGMA) L ∞ distance hierarchical clustering. Flat clusters were formed based on a cophenetic distance constraint derived from a mass and retention time tolerance which were 2 Da and 1 min respectively. Clones within a flat cluster were considered identical between runs. Hierarchical clustering of samples was performed using correlation distance and UPGMA average linkage, visualization of the tree was made using iTOL.

### Analysis of IgA1 clones shared between milk and serum using ETD data

Raw ETD spectra were used for in-depth comparison of abundant clones possibly shared between human milk and serum shown in Fig. 3. Replicate scans containing ETD spectra were grouped and summed per patient and per clone. First, precursor charge states were determined by matching precursor *m/z* to *m/z*’s of clone components detected with BioPharmaFinder. Scan grouping was done then based on the average mass and retention time, with absolute mass tolerance of up to 5 Da and retention time tolerance of up to 2 min. Summed raw profiles were saved as.mgf and analyzed further in Python. To determine the Pearson correlation, peaks in the mass-range of 1000 to 5000 m/z were selected and binned to 0.1 m/z bins. To reduce the influence of noise when calculating this correlation, each spectrum was split into 100 *m/z* slices and only the top 20 most abundant peaks in each slice were used to determine the Pearson correlation coefficients.

## Supplementary information

Supplemental Figure Legends

Supplemental Tables

Figure S1

Figure S2

## Data Availability

The mass spectrometry proteomics data have been deposited to the MassIVE repository with the dataset identifier MSV000088915.
