# Supplementary material for: Identification of common and distinct origins of human serum and breastmilk IgA1 by mass spectrometry-based clonal profiling
Source: Cell Mol Immunol. 2022 Nov 29;20(1):26–37. doi: 10.1038/s41423-022-00954-2 (PMC9707141; doi:10.1038/s41423-022-00954-2)
Supplement: Supplementary file 1 — Supplemental Figure Legends [file 41423_2022_954_MOESM1_ESM.docx]

Supplemental Figure Legends

**Supplemental Figure 1 | Mirrored top-down ETD MS/MS spectra of non-matching clones.**​

For clones A, B and C fragmentation spectra in serum (red) and milk (blue) are compared. The Pearson correlation value (r) is given for each comparison. For spectra on the right side a zoom-in is depicted to illustrate that peak patterns do not align between the scans resulting in low correlation values.​

**Supplemental Figure 2** | **SEC fractionation confirms lack of monomeric IgA1 clones in human milk.** ​

SEC fractionation of one donor across 5 time points, where each fraction was analyzed for its protein content by shot-gun proteomics. The horizontal axis depicts each analyzed fraction, and the y-axes depict the normalized abundance of the proteins, where the IgA1, IgM, IgA2 and J-chain abundances are depicted by the left y-axis and the pIgR abundance is shown by the right y-axis. Two elution peaks are observed for IgA1 and both correlate with elution of J-chain, indicating that all IgA1 assemblies observed in milk are J-chain coupled and thus that no IgA1 monomers are present in human milk.​
