# Supplementary material for: Identification of common and distinct origins of human serum and breastmilk IgA1 by mass spectrometry-based clonal profiling
Source: Cell Mol Immunol. 2022 Nov 29;20(1):26–37. doi: 10.1038/s41423-022-00954-2 (PMC9707141; doi:10.1038/s41423-022-00954-2)
Supplement: Supplementary file 2 — Supplemental Tables [file 41423_2022_954_MOESM2_ESM.docx]

| **Donor ID** | **Age**  **(years)** | **Delivery (week)** | **First sample collection**  **postpartum (days)** | **ΔCollection 1 (days)** | **ΔCollection 2 (days)** | **ΔCollection 3 (days)** |
| --- | --- | --- | --- | --- | --- | --- |
| D1 | 30 | 38 | 229 | 1 | 1 | 16 |
| D2 | 35 | 38 | 30 | 5 | 3 | 10 |
| D3 | 27 | 32* | 24 | 0 | 1 | 0 |

**Supplemental Table S1 | Maternal donor demographics and sample information.**

Notes: ΔCollection days indicate the time difference between collecting milk and serum samples. *Donor D3 was considered to have a preterm delivery (< 36 weeks gestation).

| **Protein** | **Protein Backbone Mass (kDa)** | **Average Glycosylation Mass (kDa)** | **Approximate Theoretical Mass (kDa)** |
| --- | --- | --- | --- |
| **Monomeric IgA1** | 148.5 | 12.9 | 161.4 |
| **Monomeric IgA2** | 146.2 | 17.6 | 163.8 |
| **J-chain** | 15.6 | 2.0 | 17.6 |
| **SC** | 65.3 | 8.5 | 73.8 |

**Supplemental Table S2 | Theoretical masses of IgA containing assemblies.**

Protein masses of monomeric IgA1 and IgA2 were determined by in-silico recombination of IMGT gene segments (1, 2). J-chain and SC/pIgR masses were derived from their UniProt sequences (3).
Average glycosylation masses in serum were estimated from serum glycomics data of Plomp *et al* (4)*.*
Average glycosylation masses in milk of J-chain and SC PIgR were estimated from Zhu *et al* (5)*.*

**Supplemental Table S3 | Comparison of theoretical estimated mass and experimental mass determined by mass photometry for observed IgA containing complexes.** As in these analyses total IgA is analyzed (i.e., IgA1 and IgA2) the theoretical masses are provided for both subclasses. Notably, proteomics data revealed that IgA2 is 5-50 times less abundant than IgA1 in the blood and milk of all donors (see Supplemental Table S4)

| **Name** | **IgA** | **J-chain** | **SC PIgR** | **Source** | **Theoretical (kDa)**  *IgA1/IgA2* | **Measured (kDa)** |
| --- | --- | --- | --- | --- | --- | --- |
| **Monomeric IgA** | 1 | 0 | 0 | Serum | 161.4/163.8 | 156.9 ± 17.8 |
| **Dimeric J-chain coupled IgA** | 2 | 1 | 0 | Serum | 340.4/345.2 | 338.3 ± 29.2 |
| **Dimeric SIgA** | 2 | 1 | 1 | Milk | 414.2/419.0 | 417.5 ± 39.1 |
| **Trimeric SIgA** | 3 | 1 | 1 | Milk | 575.6/582.8 | 589.9 ± 49.5 |
| **Tetrameric SIgA** | 4 | 1 | 1 | Milk | 737.0/746.6 | 770.2 ± 48.6 |

**Supplemental Table S4 | IgA1 and IgA2 protein quantification determined by bottom-up proteomics (using DIA) in the milk and serum of the three donors**

|  |  | ***Protein quantification by label free quantification (LFQ)*** | | **% IgA2 of total IgA** |
| --- | --- | --- | --- | --- |
|  |  | **IgA1** | **IgA2** |  |
| **Milk** | **D1** | 8.91E+08 | 1.66E+08 | 16 |
|  | **D2** | 1.16E+09 | 8.71E+07 | 7 |
|  | **D3** | 6.55E+08 | 2.54E+07 | 4 |
| **Serum** | **D1** | 7.15E+08 | 8.80E+07 | 11 |
|  | **D2** | 5.53E+08 | 1.17E+07 | 2 |
|  | **D3** | 7.23E+08 | 1.30E+07 | 2 |

**References**

1. Lefranc MP, Giudicelli V, Duroux P, Jabado-Michaloud J, Folch G, Aouinti S, et al. IMGT®, the international ImMunoGeneTics information system® 25 years on. Nucleic Acids Res. 2015;43(Database issue):D413-22.

2. Lefranc MP. Immunoglobulin and T Cell Receptor Genes: IMGT(®) and the Birth and Rise of Immunoinformatics. Front Immunol. 2014;5:22.

3. UniProt: the universal protein knowledgebase in 2021. Nucleic Acids Res. 2021;49(D1):D480-d9.

4. Plomp R, de Haan N, Bondt A, Murli J, Dotz V, Wuhrer M. Comparative Glycomics of Immunoglobulin A and G From Saliva and Plasma Reveals Biomarker Potential. Front Immunol. 2018;9:2436.

5. Zhu J, Lin YH, Dingess KA, Mank M, Stahl B, Heck AJR. Quantitative Longitudinal Inventory of the N-Glycoproteome of Human Milk from a Single Donor Reveals the Highly Variable Repertoire and Dynamic Site-Specific Changes. Journal of proteome research. 2020;19(5):1941-52.
