# Supplementary figures and images for: Identification of common and distinct origins of human serum and breastmilk IgA1 by mass spectrometry-based clonal profiling
Source: Cell Mol Immunol. 2022 Nov 29;20(1):26–37. doi: 10.1038/s41423-022-00954-2 (PMC9707141; doi:10.1038/s41423-022-00954-2)

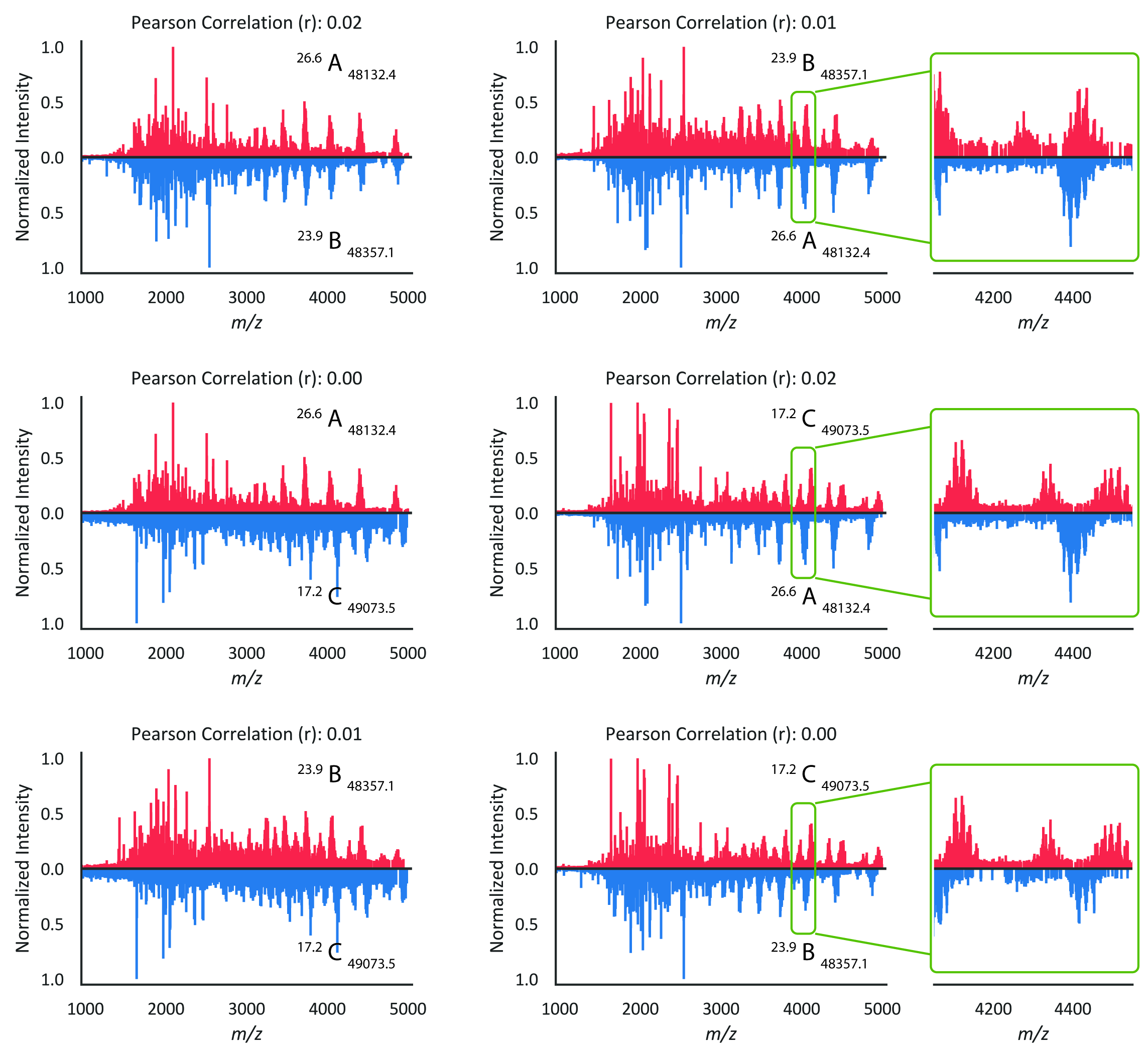

Supplement: Supplementary file 3 — Figure S1 [file 41423_2022_954_MOESM3_ESM.tif]

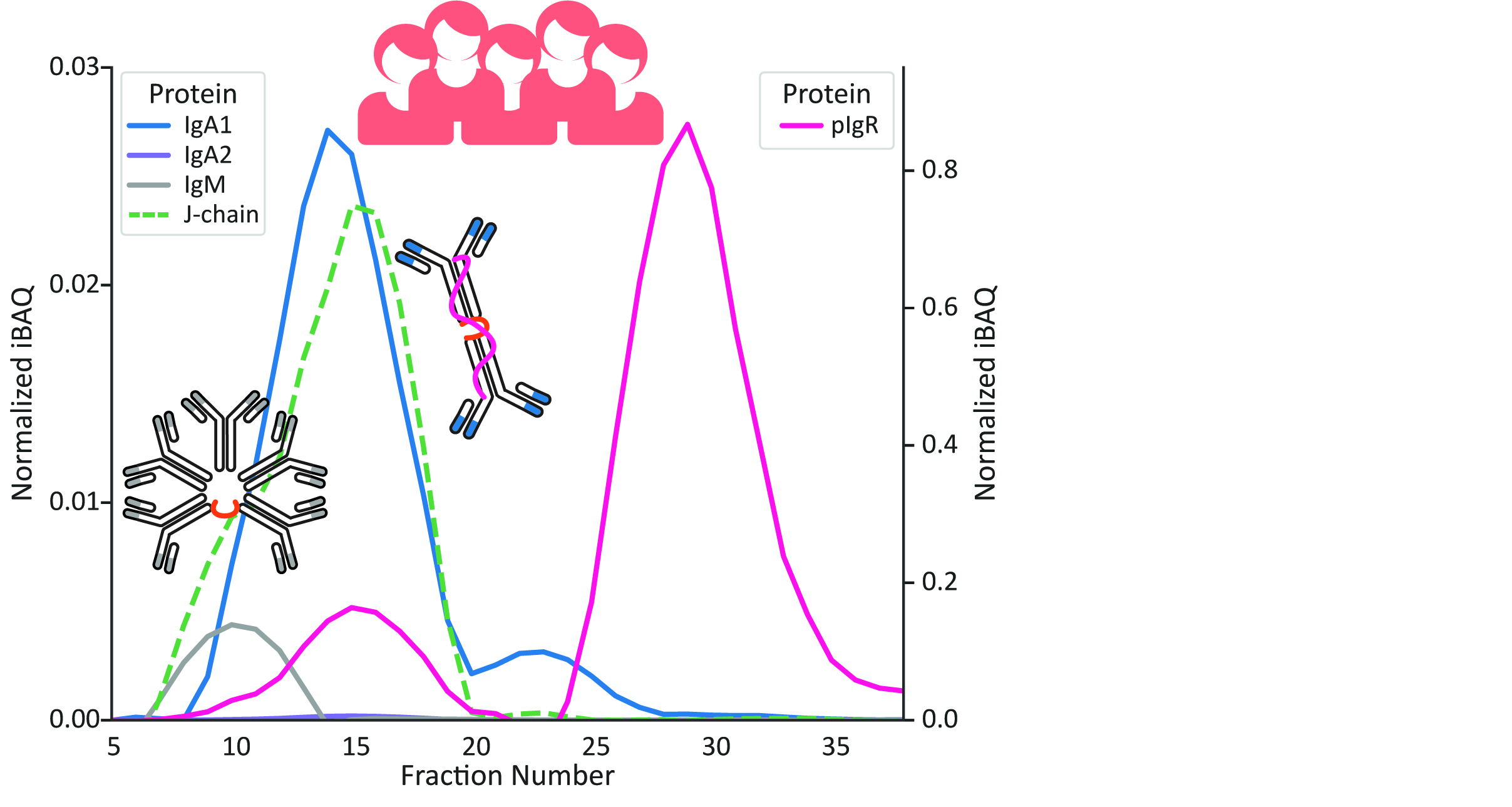

Supplement: Supplementary file 4 — Figure S2 [file 41423_2022_954_MOESM4_ESM.tif]
